# EEG Techniques with Brain Activity Localization, Specifically LORETA, and Its Applicability in Monitoring Schizophrenia

**DOI:** 10.3390/jcm13175108

**Published:** 2024-08-28

**Authors:** Angelina Zeltser, Aleksandra Ochneva, Daria Riabinina, Valeria Zakurazhnaya, Anna Tsurina, Elizaveta Golubeva, Alexander Berdalin, Denis Andreyuk, Elena Leonteva, Georgy Kostyuk, Anna Morozova

**Affiliations:** 1 Mental-Health Clinic No. 1 Named after N.A. Alekseev, Zagorodnoe Highway 2, 115191 Moscow, Russia; angelinazeltser@yandex.ru (A.Z.); savva9806@yandex.ru (V.Z.);; 2 Department of Basic and Applied Neurobiology, V. Serbsky Federal Medical Research Centre of Psychiatry and Narcology, Kropotkinsky per. 23, 119034 Moscow, Russia; 3 Faculty of Biomedicine, Pirogov Russian National Research Medical University, 117513 Moscow, Russia; 4 Institute of Biodesign and Research of Living Systems, I.M. Sechenov First Moscow State Medical University (Sechenov University), 119435 Moscow, Russia; 5 Department of Marketing, Faculty of Economics, M. V. Lomonosov Moscow State University, 119991 Moscow, Russia; 6 Department of Mental Health, Faculty of Psychology, M. V. Lomonosov Moscow State University, 119991 Moscow, Russia; 7 Department of Psychiatry, Federal State Budgetary Educational Institution of Higher Education “Russian Biotechnological University (ROSBIOTECH)”, Volokolamskoye Highway 11, 125080 Moscow, Russia

**Keywords:** electroencephalography (EEG), EEG methodologies, low-resolution brain electromagnetic tomography, schizophrenia

## Abstract

**Background/Objectives**: Electroencephalography (EEG) is considered a standard but powerful tool for the diagnosis of neurological and psychiatric diseases. With modern imaging techniques such as magnetic resonance imaging (MRI), computed tomography (CT), and magnetoencephalography (MEG), source localization can be improved, especially with low-resolution brain electromagnetic tomography (LORETA). The aim of this review is to explore the variety of modern techniques with emphasis on the efficacy of LORETA in detecting brain activity patterns in schizophrenia. The study’s novelty lies in the comprehensive survey of EEG methods and detailed exploration of LORETA in schizophrenia research. This evaluation aligns with clinical objectives and has been performed for the first time. **Methods**: The study is split into two sections. Part I examines different EEG methodologies and adjuncts to detail brain activity in deep layers in articles published between 2018 and 2023 in PubMed. Part II focuses on the role of LORETA in investigating structural and functional changes in schizophrenia in studies published between 1999 and 2024 in PubMed. **Results**: Combining imaging techniques and EEG provides opportunities for mapping brain activity. Using LORETA, studies of schizophrenia have identified hemispheric asymmetry, especially increased activity in the left hemisphere. Cognitive deficits were associated with decreased activity in the dorsolateral prefrontal cortex and other areas. Comparison of the first episode of schizophrenia and a chronic one may help to classify structural change as a cause or as a consequence of the disorder. Antipsychotic drugs such as olanzapine or clozapine showed a change in P300 source density and increased activity in the delta and theta bands. **Conclusions**: Given the relatively low spatial resolution of LORETA, the method offers benefits such as accessibility, high temporal resolution, and the ability to map depth layers, emphasizing the potential of LORETA in monitoring the progression and treatment response in schizophrenia.

## 1. Introduction

Electroencephalography (EEG) is a method for studying the electrical activity of the brain, allowing the detection of neuron signals through electrodes placed on the surface of the head. The measured voltage fluctuations in the neurons of the brain are represented in the form of sinusoidal waves, which are characterized by amplitude, power, and phase [1]. Depending on the frequency range and waveform, as well as the amplitude, topography, and type of response, EEG rhythms are distinguished, which are also denoted by Greek letters. The main rhythms are as follows:

1. Delta (δ): less than 4 Hz;
2. Theta (θ): 4–7.5 Hz;
3. Alpha (α): 7.5–12.5 Hz;
4. Beta (β): 12.5–30 Hz;
5. Gamma (γ): 30–80 Hz.

Traditional methods for analyzing EEG data include mathematical and statistical techniques to quantify frequency characteristics, such as power spectral density (PSD) estimation, coherence analysis, Fourier transform (FT), and analysis of variance (ANOVA). However, these methods have a number of limitations, such as an inability to detect changes in frequency over time and sensitivity to noise, which may result in incorrect findings [2,3].

Traditional EEG methods, although widely used in practice, do not allow us to accurately determine the spatial origin of nerve signals. This limitation is critical because the brain’s electrical activity is unevenly distributed and varies significantly across different areas [4]. General information about the electrical activity obtained using traditional methods does not provide a complete picture of the functional characteristics of specific brain structures. Thus, despite the usefulness of determining the presence of a specific frequency, understanding in which part of the brain this activity occurs is crucial for the accurate diagnosis and treatment of neurological and psychiatric diseases [5,6,7].

To overcome these limitations, new EEG analysis methods and techniques have been developed to provide more comprehensive and detailed information. In combination with EEG, new methods such as MRI (magnetic resonance imaging), CT (computed tomography), MEG (magnetoencephalography), TMS (transcranial magnetic stimulation) [8], and NIRS (near-infrared reflectance spectroscopy) allow for detailed information about activity in the deeper layers of the brain, offering greater spatial resolution [9,10,11]. Progress in the development of EEG techniques continues too, providing a variety of both invasive and non-invasive methods such as intracranial EEG (iEEG—intracranial electroencephalography), amplitude-integrated EEG (aEEG—amplitude integrated electroencephalography), and stereotactic EEG (SEEG—stereoelectroencephalography).

When using EEG, it is important to select not only the most suitable method and research technique, but also the appropriate analysis method. One of the new approaches is an improved connectivity analysis through graph theory. This approach models the brain as a network of interconnected nodes (vertices) and edges: each node represents a specific brain region or electrode site, and the edges represent the functional or structural connections between them [12,13]. Analysis based on a graph theory helps us to understand the vulnerability to damage and the functions of brain networks, providing new opportunities for research and clinical applications [14].

One of the most significant advancements that allows us to determine the functional activity of various brain structures is low-resolution electromagnetic tomography (LORETA). LORETA pinpoints brain electrical activity by creating three-dimensional maps that help to identify the source of EEG signals. Although LORETA produces somewhat blurry images due to its low spatial resolution, it allows for a significantly better localization of brain activity compared to traditional methods [15]. This method provides the possibility to accurately detect simultaneous activity in multiple brain areas and is quite easy to use due to the availability of various software packages for implementation [16]. Moreover, the level of localization errors can be reduced by using exact LORETA (eLORETA) [17].

LORETA has built-in algorithms to reduce artifacts from eye movements and muscle activity, which improves data accuracy [18]. This method not only helps to localize the activity sources, but also allows for the analysis of functional connections between different brain areas [19]. Moreover, current distribution throughout the entire volume can be measured with an improved LORETA-based method—standardized LORETA (sLORETA). By providing valuable information about the brain’s structural and functional organization, advanced techniques such as low-resolution electromagnetic tomography can be used in the diagnosis, analysis of pathogenesis, and response to therapy for significant mental illnesses such as schizophrenia. That is why, in this review, we explore both different approaches to EEG analysis and their application in studies of mental and neurological disorders.

EEG can be used for the Alzheimer’s and Parkinson’s diseases, attention deficit disorders, autism spectrum disorders, etc. [20,21,22,23,24]. Moreover, traditional EEG techniques and new approaches such as LORETA show great perspective in schizophrenia studies. EEG identified higher theta, delta and beta frequencies [25,26], decreases in the amplitude of the auditory P300 (event-related capacity component) [27,28,29], and impaired EEG activity during task performance, which confirms cognitive deficits in areas such as working memory, concentration, and executive function [30,31,32,33] in patients with schizophrenia. Violations recorded on the EEG can sometimes be detected before the full development of the disease. This potential makes EEG a valuable tool for early diagnosis [25,34] and assists in selecting the most effective treatments for patients with mental disorders [11,27].

With the help of new methods, it is possible to study brain activity in more detail in various contexts, enhancing our understanding of the mechanisms of thinking, perception, and behavior. For example, these advanced techniques can be used to classify schizophrenia [35,36,37,38] or may indicate the effectiveness of treatment and help in adjusting therapeutic strategies for a particular patient, allowing for quick modifications in the treatment approach [39,40,41,42,43].

Therefore, EEG serves as a valuable instrument for the supplementary diagnosis and surveillance of both neurological and mental disorders. In this review, we thoroughly investigated several techniques for EEG analysis, focusing on their characteristics and potential applications in the study of damage and illness, particularly concerning schizophrenia.

## 2. Materials and Methods

### 2.1. Search Strategy

For the first part of the study, which included a review of the diversity of EEG techniques, the PubMed database was selected. The literature search was performed using the following keywords “neuroimaging integration AND (MRI OR tomography OR EEG) AND cerebral localization AND local brain activity.” The search covered publications from 2018 to 2023.

For the second part of the present review, we included studies based on the LORETA implementation in schizophrenia research from the PubMed database. The following keywords were utilized: LORETA AND schizophren*. The search covered publications from 1999 to 2024.

### 2.2. Inclusion and Exclusion Criteria

Studies were included in the review if they met the following criteria:

1. Conducted with human participants;
2. Used EEG as the primary experimental method.

Studies were excluded if they met any of the following criteria:

1. Written in languages other than English or Russian;
2. Classified as reviews.

The primary selection was based on titles and abstracts. The final sample consisted of 65 articles for the first part and 29 articles for the second.

## 3. Results

### 3.1. EEG Techniques with Localization of Brain Activity

After conducting a bibliographic search, 65 studies using EEG techniques were included in the review, involving a total of 1833 male and female subjects. The analysis revealed that 33 studies included only healthy participants, while the remaining 26 studies with complete methodological data included individuals with various conditions such as epilepsy (*n* = 10, including 7 studies on the drug-refractory form), attention deficit hyperactivity disorder (*n* = 2), Parkinson’s disease (*n* = 2), tinnitus (*n* = 2), spinocerebellar ataxia (*n* = 1), laryngeal dystonia (*n* = 1), schizophrenia (*n* = 1), mild Alzheimer’s disease (*n* = 1), multiple system atrophy (*n* = 1), traumatic brain injury (*n* = 1), visual field defect due to lesions to the left/right posterior cortices (*n* = 1), autism spectrum disorder (*n* = 1), and 2 studies involved newborns.

A total of 14 studies included fewer than 20 participants. Almost half of the studies used (*n* = 29) conjugate methods such as MRI (magnetic resonance imaging), CT (computed tomography), MEG (magnetoencephalography), TMS (transcranial magnetic stimulation) [8], and NIRS (near-infrared reflectance spectroscopy). For instance, preoperative T1-weighted MRI and postoperative CT were applied to localize implanted electrodes [44,45]. When integrating EEG and MRI, it is imperative to use fMRI spatiotemporal analysis, especially when the source of interest lies deeper than EEG recording alone can capture [46]. Integrating MEG helps to measure magnetic fields and allows for a more detailed assessment of wave activity, especially in deep layers, providing a greater spatial resolution. For example, Galinsky et al. [47] used 248 magnetometers and 23 reference channels versus 32 or 64 head EEG sensors and 1 reference with a spatio-temporal resolution of 2 mm × 2 mm × 2 mm × 2 s and 80 × 95 × 75 voxels with 237 time points. In addition, fNIRS was recorded simultaneously with EEG to reduce the influence of artifacts related to muscle movement [48]. Combined hemodynamic and metabolic data obtained from bNIRS neuroimaging can enhance our understanding of brain function by providing additional information that is not as apparent when considering the EEG signal alone [49]. For example, the NIR spectrum of the brain tissue contributes to the oxygenated/deoxygenated hemoglobin concentration and the redox state of cytochrome c oxidase, the final enzyme of the electron transfer chain in mitochondria.

In the context of variations in the EEG technique, both implanted and external skin electrodes were used in the studies. The number of channels also varied, for example, taking values of four (C3, P3, C4, and P4) and eight (C3, C4, P3, P4, O1, O2, Cz, and Fz) [50,51] when participants were neonates. In 17 papers, researchers deployed 64 sensors; 128 sensors were utilized in 10 studies; and 32 electrodes were employed in 6 studies, representing the most frequent variants. Various methods were used to record the electrical activity of the brain, including iEEG (*n* = 3), aEEG (*n* = 2), and SEEG (*n* = 2). iEEG is an invasive technique that involves implanted electrodes subdurally or inside the brain, providing a high spatial resolution. In one study using iEEG, changes in the power of gamma and alpha rhythms were observed in the hippocampus in processes related to memory [52]. aEEG involves processing recorded signals to obtain amplitude-integrated curves and is commonly used in neonatology, although it has limited resolution. Using aEEG, variations in the volume of deep gray matter in premature infants were shown [50]. SEEG involves stereotactic electrode deposition methods, which allow three-dimensional mapping. This method is particularly useful for identifying epileptogenic zones. Thus, it has been demonstrated that SEEG can identify which areas affect illusions and hallucinations in drug-refractory epileptic patients [45].

Of the 65 studies we selected, methods based on graph theory were used in 13 articles.

A total of 463 participants were included in these studies. In total, nine articles had only healthy participants, and four articles compared data between a healthy control group and patients with different diseases. The experiments were aimed at finding similarities and differences between healthy people and people with diseases. Specifically, Li et al. [48] conducted a study with patients with Alzheimer’s disease (AD), including 8 healthy participants and 6 people with AD; Vikram Shenoy Handiru et al. [53] studied traumatic brain injury (TBI), including 17 chronic TBI participants and 15 healthy people; Jessica Gallina et al. examined 14 participants with lesions of the posterior cerebral cortex on the left and 9 healthy people in the control group, 13 participants with lesions of the posterior cerebral cortex on the right, and 14 healthy people [54]; and Tanu Wadhera et al. studied autism spectrum disorder (ASD), including 30 participants with this diagnosis and 30 participants who were typically developing (TD) as a control group [55].

Low-resolution brain electromagnetic tomography (LORETA) was used in 11 experiments as an analytical method for measuring EEG. Of the 11 articles, 8 studies used standardized LORETA (sLORETA) and 3 studies used exact LORETA (eLORETA). Other approaches were used in 28 articles. sLORETA—the improved LORETA-based method—is based on calculating the current distribution throughout the entire volume of the brain. In this method, with minimal localization errors, it visualizes the localization of deep sources. eLORETA allowed the researchers to reduce the level of localization errors from 12 to 7 mm [17].

In EEG research, the most common frequencies and their oscillations in experiments are as follows: delta: <4 Hz; theta: 4–7.5 Hz; alpha: 7.5–12.5 Hz; beta: 12.5–30 Hz; gamma: 30–40 Hz [56]. Of the six articles that indicated the choice of frequencies, the alpha frequency was selected in three studies, the theta frequency was selected in three experiments, the beta frequency was selected in one study, delta was selected in two experiments, and the gamma frequency was selected in two studies.

The authors of one study considered the differences in the individual ability to adapt to behavioral features reflected in the activity of brain networks at rest and they found that in the resting state, passive coping individuals are strongly involved in processes in the delta range. A limited number of areas limited by the temporal lobe in the theta and alpha ranges were also identified in subjects using active coping strategies [57].

Several studies provided insights into different aspects of functional connectivity using eLORETA and it was revealed that by stimulating the activity of the mirror neuron system, visual reproduction of its actions enhances the functional integration of this zone with the sensorimotor cortex of the brain [58].

To study the reliability of estimates of current source density (CSD) using eLORETA, an experiment was conducted on a model of sustained pain and on a model of pain-free stimulation. CSD is selected from frequencies in specific areas of the brain, called regions of interest (ROIs), in this case the anterior cingulate cortex (ACC), the anterior insula (AI), and the primary somatosensory cortex (SI), using the theta, alpha, and gamma frequency bands. The results showed that the CSD has no direct connection with the chosen area of interest and does not depend on it [59].

In one of the studies, where sLORETA was used to study the neurophysiology of event file processing, it was found that the amplitude of the wave is an event-related potential (ERP) component; the P3 component decreased during the transition from conditions without overlap to a complete overlap of features in the inferior parietal cortex (BA40), in the superior frontal gyrus (BA6), and in the medial frontal gyrus (BA9) [60]. Other data from studies aimed at studying tinnitus have revealed that in the tinnitus group, the ERP amplitude (P300) over the pregenual anterior cingulate cortex/ventromedial prefrontal cortex (pgACC/vmPFC), the dorsal anterior cingulate cortex (dACC), the posterior cingulate cortex (PCC), the primary auditory cortex, and the parahippocampus was reduced and the amplitude in the frontal and parietal regions was increased, using the sLORETA and auditory oddball paradigm [61]. Studying the cortical processing of multisensory information and integration processes in the neocortex during the experiments using sLORETA, it was presented that the activity in BA40, an area related to spatial attention and reorientation, decreased when the head position did not match the eye position or direction of movement [62]. In a study that examined the effects of ADHD (attention deficit hyperactivity disorder) on cognitive control using sLORETA, it was found that patients with ADHD show lower behavioral analysis results compared to the control group: more mistakes were made and there were long waiting periods with stimuli that indicated stopping or changing actions [63]. The authors of this study studied sensorimotor integration and differences in the primary (SI) and secondary somatosensory (SII) cortex of the brain, analyzed behavioral data using sLORETA, and were able to identify the SI and SII brain regions which differ in their ability to trigger inhibition processes [64]. In this investigation, the effect of anxiety on performance was studied using sLORETA and it was revealed that the accuracy of reactions is impaired in an anxious state in the Stroop task and error-related neural processes, characterized by reduced activation of the dorsal anterior cingulate cortex and compensatory activation in the right lateral prefrontal cortex. The Stroop task included colored words: the subjects were asked to name the colors of the written words by pressing a button with a certain sticker of this color. Half of the words written in one color coincided with the written word itself (for example, the word “red” was written in red ink); in the other half of the words there was a discrepancy between the ink and the written color itself (for example, the word “red” was written in green ink) [65]. The authors of this study focused on studying the role of the cerebellum in speech production and differences in the activity of the cerebral cortex in auditory motor regulation in spinocerebellar ataxia (SCA) patients and healthy people. During the experiment, sLORETA was used and it was shown that a meaningful, rather large vocal compensation for pitch disturbances was observed in patients with SCA compared to the control group [66]. In this study using sLORETA, the topic of cognitive flexibility and task switching processes was studied, and the results showed that at the behavior level, persistent changes in task switching were observed, which depended on the requirements for working memory, and the switching effects were more significant in the block where memory activation was required, compared to the block where it was not necessary [67]. The characteristics of the included studies are shown in Table S1: Application of LORETA for the study of brain activity in schizophrenia for diagnostic and research purposes.

Following a bibliographic search, 29 studies were examined that utilized the EEG and sLORETA (low-resolution brain electromagnetic tomography) techniques and included a total of 1904 individuals, specifically 1085 males and 764 females. Among these, 861 individuals with various degrees of previously diagnosed schizophrenia participated in the analysis across 22 trials. Moreover, the authors performed seven investigations involving 120 individuals who had experienced their first psychotic episode of schizophrenia. These patients were carefully selected based on psychiatric examinations conducted by qualified doctors. Diagnoses were made using the following criteria:

1. ICD-9 or 10 (International Statistical Classification of Diseases and Related Health Problems)—diagnostic criteria for schizophrenia in the papers by Wang J. and Kleinlogel H. [68,69];
2. DSM-III (Diagnostic and Statistical Manual of Mental Disorders) in the paper by Wölwer W. [70];
3. DSM-IV in the paper by Itoh T. [71];
4. DSM-5 in the paper by Molina V. [72].

Subjects from different cities, including Osaka (DSM-III used), Naples (DSM-IV used), and Berlin (ICD-9 used), participated in the work. The review also included research that involved 42 healthy based on their scores on the MMPI-2 (Minnesota Multidimensional Personality Inventory-2) schizophrenia and/or paranoid scale. The characteristics of the included studies are shown in Table 1.

### 3.2. Alterations in Frontal Lobe Activity in Schizophrenia

Schizophrenia is widely studied in the field of neuropsychiatry. Neuroscientists have found that the frontal lobe of the brain is often affected in people with schizophrenia. However, there is still a lack of a clear understanding of exactly how this lobe is impacted in the pathophysiology of the condition.

A review by Mubarik et al. [97] concluded the importance of frontal lobe alterations in schizophrenia. The present review included 29 articles of which 15 were noted changes in the frontal lobe. The frontal lobe contains centers responsible for conscious movement, as well as the ability to write and speak. In the analysis of the papers reviewed, several authors noted that when the P300 wave, consisting of two distinct peaks, P3a and P3b, was used, there was a reduction in the activity of the P3B source in certain brain regions. Patients with schizophrenia showed lower activity in the P3b source in bilateral frontal structures and the cingulate gyrus.

Bachiller et al.‘s study found that P3b activation in the medial frontal and anterior cingulate gyri (BA9, BA32, and BA33) and the lateral prefrontal gyrus (BA9, 10, and 11), as well as the orbital frontal gyrus (BA11 and BA25), was reduced in patients with schizophrenia [73]. No significant differences in P3a were found by Molina et al. [72]. Their study also found a reduction in the activation of the medial prefrontal regions, which was associated with widespread dysfunction across cortical networks, consistent with hierarchical models of information processing in schizophrenia.

The study by Takahashi et al. also showed a reduced activity of these medial prefrontal regions, as well as P3a-related cortical network dysfunction, which is consistent with the cognitive impairments observed in patients with schizophrenia [77]. An important factor is also a significant decrease in the level of P300 signaling compared to healthy individuals. The current density of the P300 in specific brain regions is negatively correlated with the overall psychopathology, as measured by the PANSS (Positive and Negative Syndrome Scale for Schizophrenia) total score. Specifically, the left parieto-temporal junction and the left temporal lobe showed a significant reduction in P300 amplitude in patients with schizophrenia. Pae et al. found that these patients had a markedly reduced P300 intensity, especially in the left frontal temporal cortex and bilateral prefrontal, frontal, temporal, and parieto-occipital regions [83]. A reduced current density of the MMN signal was observed in the superior temporal gyrus (STG) and inferior frontal gyrus of patients with schizophrenia [68]. The source analysis revealed a significant decrease in the current density in the inferior frontal gyrus among patients with schizophrenia. Park et al. found that there was also a decrease in the response to various triggered potentials, suggesting that schizophrenia may be related to impaired activity in the fronto-temporal-parietal network [84]. These findings could explain the cognitive and perceptual impairments seen in this patient group.

EEG using induced potentials and LORETA could be used as an additional tool to detect decreased electrical activity in the brain. There is a decrease in metabolic activity in the frontal regions of the brain, which can lead to diminished attention in individuals with schizophrenia. According to Veiga et al., many schizophrenia patients exhibit changes in the frontal lobes, including functional, structural, and metabolic changes [75]. Mubarik et al. suggest that these changes also affect areas such as the anterior cingulate and left temporal lobes [97]. Cortical dysfunction can extend beyond these areas, affecting the entire brain and showing another peak in left temporal lobe activity. Some authors, such as Mientus, note that changes in the frontal lobe are specific to chronic schizophrenia and that the prefrontal regions of first-episode patients are not damaged [96]. However, the previously mentioned alterations in prefrontal function can be associated with gradual deterioration in chronic patients, according to Kleinlogel [69]. In the general analysis of electrical activity in the brain, there has been an increase in activity in the frontal region. For example, LORETA analysis has revealed increased beta-1 activity in Brodmann area 40 (left inferior parietal lobule) and beta-2 activity in Brodmann area 10 (left medial frontal gyrus) in patients with AHs (auditory hallucinations). When comparing cerebral current density during the planning and maintenance phases, using LORETA, we found higher activation in the dorsolateral prefrontal cortex, medial frontal gyrus, cingulate gyrus, and inferior parietal cortex in healthy controls during planning compared to maintenance. However, during the PFs (planning fixations), patients with schizophrenia showed decreased neural activity in the dorsolateral prefrontal cortex (DLPFC), dorsal anterior cingulate cortex (ACC), and inferior parietal lobe. This was followed by a bilateral decrease in activity in the precentral gyrus, insular cortex, medial frontal lobe, and frontal inferior lobe [70]. The work of Mulert et al. confirmed the presence of electrical disturbances in ACC in schizophrenia, reporting an elevation in LORETA values of the middle frontal gyrus and showing a tendency for these values to be correlated with those of the precentral gyrus [78]. Higuchi et al. also noted increased activity in the middle frontal gyrus in another article [88]. Patients showed an increase in delta-band activity, with a maximum in the left inferior temporal gyrus and the right middle frontal gyrus. Itoh et al. indicated in their work positive dynamics in areas associated with schizophrenia during treatment [71]. In addition, another study noted an increase in delta and theta frequencies in the anterior cingulate and medial frontal cortex in patients receiving clozapine and in those who had not previously taken antipsychotic drugs [92]. These results suggest that changes in activity in certain brain regions may be associated with psychopathology and may be relevant to specific treatments for schizophrenia.

### 3.3. Alterations in Temporal Lobe Activity in Schizophrenia

Of the 29 studies using LORETA, 15 found changes in temporal cortex activity in patients with schizophrenia. This suggests an important role for this area in the pathophysiology of the disorder. For example, Bachiller et al. found that delta activity was higher in the spindle gyrus region of patients with schizophrenia [73]. Itoh et al. also found increased delta-band activity in patients, with a maximum in the left inferior temporal gyrus and right middle frontal gyrus [71]. Mientus et al. in their work concluded a significant increase in delta-band activity in patients with schizophrenia compared to healthy individuals, especially in the fusiform gyrus of the temporal lobe [96]. On the contrary, another study found a decrease in activity in the left temporal lobe when listening to sounds of different tonality [74]. It is known that this brain region is involved in the processing of non-main auditory stimuli. It is likely that the left temporal lobe is associated with the pathogenesis of schizophrenia. Specifically, the left insular cortex and superior temporal gyrus have been identified as important areas involved in this process. A related study found an asymmetric location of P300 sources in these regions, which correlated with PANSS scores [68].

Furthermore, the LORETA (low-resolution electromagnetic tomography) study revealed that patients with schizophrenia also have decreased activity in other regions of the brain, such as the right inferior temporal lobe, parahippocampal gyrus, and hippocampus. These areas are known to be associated with planning, and they may explain the lower information processing ability observed in patients. Data were obtained for a notable decrease in P300 density in the left medial temporal and inferior parietal areas among patients with schizophrenia compared to a control group. Additionally, there were inconsistencies in the activation patterns of P300 in certain areas of the brain and how these patterns relate to clinical symptoms. The density of P300 in specific brain regions had a negative correlation with the overall severity of psychopathology as assessed by the PANSS total scores [83]. With regard to other sources, MMN (Mismatch Negativity) was primarily localized in the superior parietal lobe (BA7). There was a significant decrease in the current density of MMN in the left superior temporal gyrus, although this area displayed marked activation in control subjects. This may be attributed to impaired prefrontal sensory processing among patients [84]. Based on the MMPI-2 questionnaire, women with schizophrenia were divided into two groups: high and low psychotic. Highly psychotic women had a higher LORETA current density in the left frontal and temporal cortical areas, including Brodmann fields 11 and 38. This indicates greater activity during auditory stimulation [76]. In contrast, in the same oddball paradigm, patients with schizophrenia had reduced neuronal activity in the temporal cortex. Specifically, there was a reduction in the P300 current density, particularly in the left hemisphere [82].

Regarding other paradigms, training in affect recognition (TAR) is considered an important approach for improving socio-cognitive skills. After TAR sessions, event-related potentials (ERPs) were examined, and it was found that left hemispheric activity in the temporal lobe, including the spindle gyrus, decreased after 172 milliseconds [94]. In another study, patients showed a lower P300 density in the left hemisphere. However, after six months of treatment with olanzapine, an increase in this density was observed and the data were similar to those of healthy subjects. This levodominant pattern was associated with improvements in negative symptoms and verbal memory. Reduced negative symptoms and improved verbal memory were associated with increased activity of the left superior temporal gyrus (STG). Olanzapine was indicated to restore the normal pattern of neuronal activity in the temporal lobes, especially in Heschl’s gyrus. The study also found a significant reduction in beta-3 activity in the frontal temporal cortex in patients taking olanzapine compared to those not taking antipsychotics. Another type of treatment, rTMS (transcranial magnetic stimulation), after 2 weeks, affected current changes in the temporal lobes of individuals with schizophrenia. More specifically, the current density decreased in the beta-1 and beta-3 bands of the temporal lobe, depending on the side of stimulation [89]. Thus, using the LORETA method, the role of the temporal lobe of the cerebral cortex in the pathophysiology of schizophrenia has been shown. Several studies have found a decrease in neural activity, especially in the left temporal lobe and superior temporal gyrus (STG), which is associated with impaired sensory processing in patients with schizophrenia. Thus, there was an increase in delta-band activity, especially in the fusiform gyrus and inferior temporal gyrus. Cognitive impairment was associated with a decreased P300 and MMN density in the left medial temporal gyrus and superior temporal gyrus, respectively. Treatments such as medication (e.g., olanzapine) and rTMS show potential for symptom relief by normalizing P300 patterns and beta-band activity.

### 3.4. Particularities of the Alterations in Brain Activity in First-Episode Patients

Most studies measured reaction time. Analysis of the data revealed significant differences between the mean reaction time of patients who had experienced a first episode of psychosis and the control group. In all studies, there was an increase in reaction time in the experimental group of patients compared to the control group [68,69,70,74].

In the previous sections, we have described general trends in brain changes in patients who have had a first psychotic episode. Next, we will look at individual brain regions in which statistically significant changes have been found.

To our knowledge, the first description of first-episode patients in the context of the LORETA method was made by Gallinat et al. The LORETA method revealed activation in the primary auditory cortex and in the anterior cingulate region. In addition, an adapted multi-dipole model showed activation of the temporal–radial source in areas other than primary hearing. In patients with schizophrenia, a significant deficit in activation was found in the anterior cingulate and in the left secondary auditory cortex [74]. Although the findings shed light on the neurobiological abnormalities associated with schizophrenia, it is not possible to clearly differentiate between chronic and first-episode patients on the basis of the available information, as they were considered in the same comparison group.

The 2005 study by Lehmann et al. studied only first-episode patients who were not taking drug therapy. The LORETA method revealed enhanced cortical activity in patients with schizophrenia, especially in the left hemisphere. LORETA imaging also showed higher activity in the left precentral gyrus compared to controls and reduced activity in the right inferior parietal lobe. An important finding is the predominance of activity in the anterior part of the left hemisphere in patients with schizophrenia. Overall, the study showed that patients with schizophrenia have altered activity patterns in specific brain structures, including the left precentral gyrus and the right inferior parietal lobe [87].

In 2007, Kleinlogel et al. suggested that in the early stages of schizophrenia that NGA patterns would be more similar to those of healthy people than in people with chronic schizophrenia, which may indicate unique neurophysiological variations at different stages of the disorder. In summary, the study showed that patients had a reduced P300 amplitude compared to healthy subjects, especially at parietal electrodes after Go stimuli and at central electrodes after NoGo stimuli. In addition, patients with a first episode of schizophrenia showed differences in NGA (NoGo-anteriorization) scores compared to healthy subjects. The LORETA method revealed that after NoGo-stimulus in patients the source is localized in the prefrontal region, while in healthy subjects it is in the central cortex. Increased NGA in patients may indicate early changes in the prefrontal cortex [69].

Wang et al. also studied patients with a first episode of schizophrenia. Patients who were not taking therapy were selected, and the study used 60-channel recording for more accurate visualization. After visualizing the data using the LORETA method, in the control group the P300 current density was symmetrically distributed in both hemispheres: frontal, parietal and temporal lobes. In patients with schizophrenia, the P300 current density was symmetrical in the frontal and parietal lobes, but in the left temporal lobe, it was attenuated. The results obtained by Wang et al. suggest that functional changes in the left middle temporal gyrus are probably not related to drug side effects or disease duration, and thus this brain region may be involved in the pathogenesis of schizophrenia as a cause of the disease [68].

Reduced gray matter volume and impaired prefrontal cortex functionality have been observed in individuals with schizophrenia, contributing to cognitive impairment and impaired inhibitory processes [98]. Barch et al. noted that not only in patients with chronic schizophrenia, but also in patients with a first episode of schizophrenia, there is a decrease in the gray matter volume of the frontal brain structures [99]. As reported in previous sources as well, the prefrontal cortex plays a crucial role in altering EEG activity. Itoh et al., using the LORETA method, showed that negative symptoms of schizophrenia may be associated with structural abnormalities in the prefrontal cortex. In the context of EEG activity in patients with a first episode of psychosis not taking neuroleptics, an increase in delta-band activity was observed in the prefrontal cortex, namely in the right middle frontal gyrus (MFG), right inferior frontal gyrus (IFG), and right superior frontal gyrus (SFG). This increase in delta-band activity may be related to the deficits in working memory and executive function that are commonly seen in schizophrenia [71]. Another important finding in this study by Itoh et al. was that in patients with a first episode of psychosis who were not taking neuroleptics, the current density of delta activity in the left inferior temporal gyrus was negatively correlated with the severity of negative symptomatology, which in turn could be explained by decreased dopaminergic activity in these structures [71,98].

Wölwer et al. further investigated prefrontal cortex function in patients with a first psychotic episode. They hypothesized that differences between the groups manifest themselves during planning. This is because patients with schizophrenia have difficulties with cognitive flexibility and cognitive control. To correlate functionality with cognitive processes, the planning and monitoring phases during the performance of the TMT-B test were investigated. In patients who had suffered a first psychotic episode, hypoactivity in prefrontal brain regions was observed, especially during fixation on planning [70].

Areas of high interest were identified using the LORETA method. During a behavioral test during the planning phase, patients with a first episode of psychosis showed decreased activity in the dorsolateral prefrontal cortex of the DLPFC (BA46), the dorsal part of the anterior cingulate cortex (ACC) (BA32), and the inferior parietal cortex (BA40). In addition to these areas, patients with schizophrenia showed bilateral decreased activity in areas of the precentral gyrus and islet in the medial frontal gyrus (BA10) and inferior frontal gyrus (BA45). Decreased activity was also observed in lateral regions of the right hemisphere inferior temporal gyrus (BA20), parahippocampal gyrus, and hippocampus [70].

Recent data obtained by Molina et al. suggest significant changes in current density in different brain regions in patients with schizophrenia, both chronic and first-episode, compared to controls [72].

Patients with chronic schizophrenia had reduced activity in specific brain regions such as the medial frontal and anterior cingulate, middle frontal gyrus, and orbital frontal gyrus. No such activity was observed in a control group of healthy individuals. Patients with a first episode of schizophrenia also had reduced activity in the superior and middle frontal lobes of the brain. This may indicate that these brain structures become involved in cognitive dysfunction early in the disease. The control group showed increased activity in the parietal and frontal lobes. Interestingly, it showed a positive relationship between the activity level and working memory. Overall, the results of the present study suggest that areas involved in the P3b task are hypoactive with respect to structural connectivity in schizophrenia, although this hypoactivation may be exacerbated by antipsychotic medication [72].

### 3.5. The Impact of Therapy on the Recovery of Brain Activity in Schizophrenia

In total, 15 of the 29 studies involved the use of medication to treat the subjects. In five papers, authors were able to track the effect of treatment on the results; one used pharmacotherapy in combination with rTMS when obtaining images with LORETA.

There was a reduced NGA demonstrated, indicating an overall change in the brain’s electrical activity towards the prefrontal areas in individuals receiving atypical neuroleptics: six patients—clozapine, five patients—olanzapine, and one patient—risperidone. Each day, the mean neuroleptic dose was 392.1± 416.4 mg chlorpromazine equivalents [81]. The following study of electrical brain activity involved 41 people with a diagnosis of schizophrenia. Among them, 11 were antipsychotic-naive, 8 were taking clozapine, 10 were on olanzapine, and 12 were on risperidone. Based on the data, it can be observed that the temporal cortex exhibits alpha-1 and alpha-2 frequencies, while the temporo-occipital posterior limbic areas show an increase in beta-1 and beta-2 frequencies.

In another case, four patients received haloperidol treatment, three received risperidone, four received perospirone, one received chlorpromazine, and three received both risperidone and perospirone. Taking olanzapine for 6 months enhanced LORETA values in the left superior temporal gyrus. The administration of olanzapine enhanced the P300 current source density in the left superior temporal gyrus (STG), as indicated by the LORETA values. Furthermore, there was a rise in P300 current source density observed in the left middle frontal gyrus and precentral gyrus [88]. A long-term study in which patients were treated with olanzapine for 6 months showed a more significant effect. Olanzapine restored the principal neuronal activity pattern in the temporal lobes, namely in the Heschl gyri (also known as the transverse temporal gyri), according to LORETA images of P300 [90]. Furthermore, when comparing the electrical activity of the brain in olanzapine patients and those who had not previously taken any antipsychotic drugs according to LORETA images, there was a significant difference in electrical brain activity in the group of subjects. There was an observed rise in theta frequencies in the anterior cingulum and a decrease in the presence of beta-3 frequencies in the frontotemporal cortex and anterior cingulum. Moreover, there was a decline in the occurrence of alpha-1, beta-2, and beta-3 waves in the occipital brain and posterior limbic areas. After comparing individuals who received clozapine treatment with those who had not previously taken any antipsychotic medication, we observed a rise in delta and theta frequencies in the anterior cingulate and medial frontal cortex, along with a decline in alpha-1 and beta-2 frequencies in the occipital regions.

However, no significant differences were observed in the electrical activity of the brain between individuals who had not previously been exposed to antipsychotic drugs and those who had been treated with risperidone [92].

One study evaluated the impact of repetitive transcranial magnetic stimulation (rTMS) on alterations in brain metabolism while patients were receiving medication therapy. Six patients were on monotherapy (olanzapine 15 and 20 mg, levomepromazine 50 mg, quetiapine 600 mg, amisulpride 900 mg, and ziprasidone 160 mg); and three patients were on two antipsychotics (risperidone 4 mg with amisulpride 500 mg or clozapine 100 mg, and olanzapine 5 mg with haloperidol 4 mg). The last three patients were on a mix with mood stabilizers (lithium carbonate 1350 mg with sulpiride 500 mg and levomepromazine 25 mg, carbamazepine 900 mg with risperidone 3 mg and ziprasidone 160 mg, and carbamazepine 900 mg with quetiapine 800 mg and fluphenazine decanoate 40 mg every two weeks). The rTMS changed the brain metabolism in the left superior temporal gyrus and in linked areas as well as in the frontal lobes and contralateral cortex. Whereas an increase was recorded for the beta-2 band contralaterally, the authors observed a reduction in current density (LORETA) for the beta-1 and beta-3 bands in the left temporal lobe [89]. Cumulative data on the effects of drug treatment and rTMS are presented in Table 2.

It can be concluded that olanzapine and clozapine have a positive effect on the electrical activity of the brain in patients with schizophrenia. Thus, using recorded EEG data and LORETA imaging, it is noted that some antipsychotic drugs can improve cognitive and functional performance in people diagnosed with schizophrenia. It can be assumed that the use of low-resolution electromagnetic tomography is possible to assess the response to therapy and monitor the effectiveness of the treatment.

## 4. Discussion

The first part of this review included 64 studies to explore the diversity of EEG techniques and adjuncts. Approximately half of these studies used a combination of methods (MRI, CT, MEG, TMS, and NIRS) as well as variations of EEG techniques (iEEG, aEEG, SEEG). The use of additional techniques has shown that it is possible to achieve a more accurate localization of electrodes and to obtain a detailed picture of brain activity in the deep layers of the brain. Studies using the LORETA method, as well as its more accurate and reliable variations sLORETA and eLORETA, provided detailed distributions of brain activity in different frequency bands (alpha, theta, and delta most often). Thirteen articles used graph theory to analyze the data, considered a powerful method for describing the functional connectivity of brain regions [57]. While fMRI is renowned for its high spatial resolution in localization, NIRS, a method based on measuring blood oxygen levels, is a more cost-effective and convenient alternative, albeit with lower sensitivity [100]. The LORETA method addresses the inverse localization problem when the feedback signal is related to the voxel current density. However, there is an overlap between the current densities of nearby voxels and a decrease in spatial resolution, which can be mitigated in the more advanced sLORETA method [94]. The benefits of the LORETA approach are also covered in other research, emphasizing its solving of the inverse problem by employing the smoothest constraint assumption given the Laplace operator and that it does not necessitate an assumption on the number of activity sources [83]. LORETA is one of the most promising methods with several advantages and disadvantages compared to the EEG and imaging methods mentioned in the review (MRI, CT, MEG, TMS, and NIRS). Despite its relatively low spatial resolution, LORETA can create three-dimensional maps of brain activity, localize the sources of EEG signals, and identify functional connections between different brain regions. MRI, on the other hand, does not provide information on functional brain activity and provides difficulties in integration with EEG, although MRI has a high spatial resolution for visualizing brain anatomy. CT provides less soft tissue detail than MRI and also does not provide information on functional activity, but it is effective in detecting acute conditions. MEG is considered a more expensive method, but its resolution allows the assessment of neuronal activity. TMS is invasive and not as accurate for detecting activity patterns but serves as an effective therapeutic tool. NIRS is not suitable for studying activity in deep layers but is effective for continuous monitoring, although it has limited spatial resolution. Thus, LORETA is considered a more accessible method, with high temporal resolution, and does not impose contraindications for patients with metal implants and pacemakers. Due to the advantages of the LORETA method, the second part of the review focuses on this technique in more detail.

The second section of this review includes 29 studies that used the LORETA method to identify patterns in schizophrenia. Including 1904 study subjects cumulatively, these papers provide important insights into brain activity in a massive sample. Studies of schizophrenia often show asymmetry in hemispheric activity. Changes in neural activity in patients with schizophrenia are particularly demonstrated in the left hemisphere including the frontal, limbic, parietal, and temporal lobes. However, Itoh et al. have also shown increased activity in the right middle frontal gyrus. This asymmetry is associated with cognitive deficits in working memory and processivity [71]. However, other studies not using the LORETA method but involving MRI have noted marked changes in the frontal, limbic, occipital, parietal, and temporal lobes of the right hemisphere [101]. More specifically, the review highlights abnormalities in the frontal and temporal lobes. The former plays a critical role in planning and has shown reduced activity in patients in areas such as the dorsolateral prefrontal cortex and anterior cingulate cortex [70], indicating impaired cognitive flexibility. Assessment of the P3b component revealed decreased activity in the medial frontal and anterior cingulate gyrus, lateral prefrontal cortex, and orbital frontal gyrus. The observed decrease in activation correlates with deficits in cognitive abilities. In addition, a decrease in MMN current density was found in the superior temporal gyrus and inferior frontal gyrus, which proves the broad impact of schizophrenia on brain function. Regarding metabolic activity, it was also reduced in the frontal lobe, which may demonstrate reduced attention in patients. The analyzed data are consistent with previous studies. Alterations found in the temporal lobe involved in auditory processing and sensory integration, namely the left medial and superior temporal gyrus, may be associated with auditory hallucinations [68]. Several studies have observed increased delta activity, especially in areas such as the spindle gyrus, fusiform gyrus, and inferior temporal gyrus. This may reflect pathological overactivation associated with disease progression. Delta-band activity is often associated with cognitive dysfunction. The P300 component, associated with attention and cognitive abilities, showed reduced density in the left medial temporal and inferior parietal regions in patients with schizophrenia. It can be suggested that P300 may serve as a biomarker of schizophrenia severity. MMN localized predominantly in the superior parietal lobe and showed reduced current density in the left STG in patients.

The findings raise a debate based on the categorization of structural changes as a cause or as a consequence of schizophrenia. This is crucial for treatment development and an understanding of the temporal dynamics. It has been demonstrated that structural changes, including reduced gray matter volume and altered activity in frontal and temporal cortical areas, are present in patients with a first episode of schizophrenia [99]. Certain changes may likely contribute to the progression of the disease. Expanding on this theme, it has been found that chronicity and medications such as haloperidol can exacerbate these structural abnormalities, causing the atrophy of brain matter [89]. This is why it is important to compare chronic and first-episode patients to assess the extent to which the changes are progressive. Molina et al. showed the likelihood of a worsening and greater spread of structural abnormalities in the chronic form [72].

Focusing on pharmacological treatment with antipsychotic drugs such as olanzapine and clozapine revealed changes. Treatment with olanzapine for six months resulted in an increase in LORETA values in the left STG and restoration of the P300 current source density to the level of the control group. This allows us to conclude about the positive effect of the drug on neuronal activity. Clozapine stimulated an increase in delta and theta activity in the anterior cingulate and medial frontal cortex. The role of transcranial magnetic stimulation in combination with drug therapy was also studied and the positive effect of the drugs was shown to be enhanced.

A more detailed examination of the work relating to the first episode of schizophrenia reveals certain areas of the brain in which significant changes are observed. Namely, change is observed in the prefrontal cortex and temporal lobe, especially in the left middle temporal gyrus. Functional changes in these areas are thought to be able to precede structural changes [68]. These areas could potentially serve as biomarkers for early diagnosis and treatment strategies. The promising effects of antipsychotic agents on brain activity, specifically cognitive and functional patterns, have already been shown [81,88]. Some of the affected areas at the first episode are also seen in chronic disease, such as the anterior cingulate cortex and areas of the frontal cortex [70,72,87]. This suggests that these changes are associated with disease onset rather than treatment and deterioration.

The usefulness of the information analyzed cannot be overestimated; however, there are limitations in observed studies, including the heterogeneity of patient groups and treatment protocols. This may lead to differences in results. Additionally, there is indeed a proportion of patients who may have an organic psychotic disorder that manifests symptoms characteristic of schizophrenia but is of non-endogenous origin (e.g., epilepsy, neuroinfection, brain tumors, brain injury, cerebral vascular disease, or other severe diseases) and does not lead to the formation of a schizophrenic defect. There is research evidence [102] on the frequency of schizophrenia symptoms in patients with organic mental disorders. A recent study showed that 25% of patients previously diagnosed with schizophrenia were actually experiencing organic psychosis [103]. These patients often have focal changes in the temporal, parietal, and frontal lobes. There is evidence that in a study of first-episode schizophrenia, 6% of cases were associated with organic illness [104]. Therefore, we do not consider the LORETA EEG as a determinant of diagnosis because there are schizophrenia-like disorders that may be mistaken for schizophrenia. Despite the advantages of the LORETA method, its resolution is relatively low compared to other types of neuroimaging, which may affect the accuracy of localization of changes. To improve the comparability of studies, the development of a common methodology with the tracking of changes in activity over a long period is important. Combining LORETA with MEG or fMRI may increase the effectiveness of the study. Additionally, our research included articles from a single database over a limited period, not covering other localization and EEG techniques, such as the monitoring of infrared, magnetic, and electromagnetic fields. A comprehensive evaluation was conducted on articles published between 2018 and 2023, focusing on emerging EEG signal localization approaches. This was motivated by the rapid advancements in the field and the potential obsolescence of data processing techniques to identify and describe the most relevant techniques available today. When reviewing the schizophrenia data gathered by the LORETA approach, we did not need to reduce the time interval. Our goal was to obtain a comprehensive understanding, so it was essential to utilize all the data on the functional changes in the brain that had been collected.

Thus, despite the relatively low spatial resolution provided by LORETA, with the availability of more advanced localization techniques such as fMRI, LORETA has advantages in the study of schizophrenia patterns. Above all, the affordability of LORETA, which maps deep brain layers and allows us to focus on structural and frequency-dependent changes, is a decisive factor. Also valuable is the ability to process large amounts of data on the dynamic changes needed to monitor disease progression or the response to schizophrenia treatment.

## 5. Conclusions

The study was largely aimed at identifying the variety of EEG techniques and their combinations, including the use of LORETA and the improved sLORETA and eLORETA techniques. This allows a more accurate localization of brain activity sources in different frequency ranges. The study emphasizes the importance of integrated approaches for studying changes in functional connectivity, especially in schizophrenia. This work adds to the existing knowledge about the diversity of EEG and neuroimaging techniques, the detection of activity changes in different brain regions, and the effect of antipsychotic drugs on neural activity visualized using the methods listed in the review. Incorporating LORETA may be promising for monitoring disease progression and evaluating treatment.

## Figures and Tables

**Table 1:** Characteristics of the included studies. (SCZ—schizophrenia, CTL—healthy controls, dfSCZ—drug-free patients with schizophrenia, H—healthy participants, BPE—brief psychotic episode, SCZt—schizotypal personality, D—unmedicated depressive patients, DS—depressed menopausal syndrome).

Study	Date	Sample Size	Population Characteristics	Drug Therapy (S)	Methods	Paradigms	EEG Sensors (Scalp)	Results
Bachiller et al. [73]	2015 Dec	SCZ = 31, CTL = 38	S: 36.25 ± 9.62 years, (M:F) = 21:10; C: 33.35 ± 12.26 years, (M:F) = 23:15	Atypical antipsychotics	ERP (N200, P300), sLORETA	3-stimulus oddball paradigm	17 (Fp1, Fp2, F3, F4, C3, C4, P3, P4, O1, O2, F7, F8, T5, T6, Fz, Pz and Cz)	Over the superior and medial frontal gyrus (BA10, BA11), orbital frontal gyrus (BA11), bilateral anterior cingulate gyrus (BA33), and cingulate sulcus (BA31, BA32, BA25), P3b source activity was lower in the SCZ group.
Gallinat et al. [74]	2002 Sep	dfSCZ = 21, CTL = 21	dfS: 30.0 ± 9.8 years, (M:F) = 14:7; CTL: 30.0 ± 8.6 years, (M:F) = 14:7	Drug-free	ERP (N1), LORETA	Listening to 230 sounds of different pitches (1000 and 2000 Hz)	32 (with the additional electrodes FC1, FC2, FC5, FC6, T1, T2, CP5, CP6, PO9, and PO10)	The mean reaction time of patients was higher. In individuals with SCZ, the amplitude of N1 in Cz was significantly smaller. A decrease in N1 amplitude in persons with SCZ was observed in the area of temporal electrodes, predominantly on the left side.
Veiga et al. [75]	2003 Sep	SCZ = 25, CTL = 40	S: 29.6 ± 7.98 years, (M:F) = 16:9 females; CTL: 30.4 ± 7.99 years, (M:F) = 23:17	Antipsychotics/typical or atypical neuroleptics	LORETA, fast Fourier transform (FFT)	Eyes-closed alert/resting	19 mono-polar	SCZ only varied substantially from CTL during resting conditions in frontal regions at the delta–theta frequency range.
Mucci et al. [76]	2005 Jul 30	CTL = 42	22.9 ± 2.6 years, (M:F) = 17:25	Drug-free	ERP (P300), LORETA, neuropsychological evaluation	Three-tone auditory oddball paradigm (P300)	32	In highly psychotic women, a higher current density was shown in the left frontal and temporal regions, including BA11 and 38, compared with women with a low level of psychosis, and P3a activity was shifted to the left.
Takashi et al. [77]	2013 Feb 1	SCZ = 410, CTL = 247	S: 45.6 ± 8.9 years, (M:F) = 299:111; CTL: 43.2 ± 11.4 years, (M:F) = 118:129	Second-generation antipsychotics/first-generation antipsychotics/combination/drug-free	eLORETA, ERP (P3a, MMN)	Auditory oddball paradigm	34 (Fp1, Fp2, Fz, F3, F4, F7, F8, FC1, FC2, FC5, FC6, Cz, C3, C4, CP1, CP2, CP5, CP6, Pz, P3, P4, P7, P8, O1, O2, PO9, PO10, Iz, T1, T2, T7, T8, TP9, and TP10)	SCZ observed significant reductions in MMN and P3a responses in the temporal, frontal, and parietal regions. These abnormalities may have resulted from impaired orientation or shifting attention to infrequent stimuli.
Wölwer et al. [70]	2012 Jul 30	SCZ (first episode patients) = 12, CTL = 12	S: 32.2 ± 8.2 years, (M:F) = 8:4; CTL: 30.1 ± 5.8 years, (M:F) = 8:4	Not stated	LORETA	Trail-making test (TMT)-B	32	Significant correlations were found between task performance and brain current density, especially in the case of CTL. In SCZ, these correlations had a similar character, although they were significantly weaker.
Mulert et al. [78]	2001 Apr	SCZ = 18, CTL = 25	S: 36.27 ± 11.04 years, (M:F) = 7:11; CTL: 34.16 ± 10.99 years, (M:F) = 13:12	Drug-free	ERP (N1), LORETA	Selective attention tasks and willed action paradigms	19	The mean reaction time was increased in the group SCZ. The average error rate was higher in SCZ patients.
Wang et al. [68]	2010 Mar	dfSCZ (first episode) = 19, CTL = 25	S: 28.63 ± 12.33 years, (M:F) = 12:7; CTL: 32.88 ± 9.39 years, (M:F) = 12:13	Drug-free	LORETA, ERP (P300)	Auditory oddball paradigm	60 (FP1, F7, FP2, F3, FC3, FT7, T7, F8, F4, Fz, FCz, C3, TP7, FT8, FC4, Cz, CPz, CP3, P3, P7, T8, TP8, C4, P8, CP4, P4, Pz, Oz, O1, O2, FPz, AF3, AF7, F5, AF8, AF4, F1, FC5, F6, F2, FC1, C5, FC6, FC2, C2, C1, CP1, CP5, P5, PO7, PO8, C6, CP6, P6, CP2, PO4, P2, POz, P1, and PO3)	In individuals with SCZ, the button press accuracy was reduced and mean reaction time was increased. The decrease in P300 amplitude in patients occurred predominantly in the left hemisphere. The effect of LMR was significant only for the control group.
Neuhaus et al. [79]	2007 Oct	SCZ = 16, CTL = 16	SCZ: 36.25 ± 8.4 years, (M:F) = 8:8; CTL: 36.63 ± 8.1 years, (M:F) = 8:8	Atypical antipsychotics/selective serotonin reuptake inhibitors/flupentixol decanoate	LORETA, ERP (N200, P300)	Attention network test (ANT)	32 (with the additional electrodes FC1, FC2, FC5, FC6, T1, T2, CP5, CP6, PO9, PO10, and Lo1)	In the conflict scenario, P300 latency increased at Cz and the P300 amplitude decreased at Pz. An anterior cingulate cortex deficit was detected.
Sabeti et al. [80]	2011	SCZ = 20, CTL = 20	No data	No data	LORETA	Oddball paradigm (P300)	No data	The most active area in the context of P300 in a group of normal individuals is the cingulate gyrus, one of the key elements of the working memory circuitry. The frontal lobe is the most active region in the SCZ group and the source of P300.
Fallgatter et al. [81]	2003 Sep 30	SCZ = 31, CTL = 31	SCZ: 30.3 ± 8.6 years, (M:F) = 22:9; CTL: 30.7 ± 3.5 years, (M:F) = 22:9	Typical or atypical neuroleptics (clozapine, *n* = 6; olanzapine, *n* = 5; and risperidone, *n* = 1)/benzodiazepines/antidepressants/carbamazepine/valproate/lithium/biperiden/drug-free	LORETA	Continuous performance test (CPT)	21 (Fp1, Fp2, F3, F4, F7, F8, T3, T4, C3, C4, T5, T6, P3, P4, O1, O2, Fpz, Fz, Cz, Pz, Oz)	Patients with SCZ had longer reaction times and also made more errors of omission. The mean NGA was significantly lower in patients with SCZ. In five patients who were not taking neuroleptic medication, NGA was significantly reduced compared to the control group, but did not differ from patients taking neuroleptics. Activity in the anterior cingulate fossa was significantly weaker in patients with SCZ.
Wang et al. [82]	2003 Mar	SCZ = 13, CTL = 20	SCZ: 32.3 ± 10.6 years, (M:F) = 13:0; CTL: 34.6 ± 8.4 years, (M:F) = 20:0	All medicated	LORETA, ERP (P300)	Oddball paradigm	128	A dramatic decrease in total P300 current strength was found in SCZ patients. The P300 current strength was higher on the right than on the left, in the inferior temporal region. The P300 current density in the patient group was consistently lower and mainly concentrated in the left hemisphere, especially in the left fronto-temporal cortex.
Pae et al. [83]	2003 Nov	SCZ = 20, CTL = 21	SCZ: 29.2 years, (M:F) = 14:6; CTL: 26.5 years, (M:F) = 19:3	Chlorpromazine	LORETA, ERP (P300)	Oddball paradigm	128	P300 amplitude was lower in the group SCZ than in CTL. The P300 sources in the group S were less focused in the superior parietal region and extended to the left prefrontal and left temporal regions, while CTL P300 sources were localized in the left superior parietal region.
Park et al. [84]	2002 Nov	SCZ = 14, CTL = 14	SCZ: 26.5 years, (M:F) = 9:5; CTL: 24.6 years, (M:F) = 9:5	Chlorpromazine	LORETA, ERP (MMN)	Eyes fixed on a single point, listening to the sounds	128	There were many MMN generators in the left hemisphere of control participants, including the inferior frontal gyrus, superior parietal lobe, STG, and IPL. The majority of MMN sources in patients with SCZ were found in the superior parietal lobe (BA7), with no consistent sources observed in the left IPL and left STG.
Gornerova et al. [85]	2023 Jan 18	SCZ = 19	SCZ: (M:F) = 11:8	Chlorpromazine/clozapine/anticonvulsants/benzodiazepines	sLORETA, LF-rTMS	10 days of monitoring	No data	Shows changes in EEG current density and lag-phase synchronization in beta- and alpha-2 bands during treatment of patients with auditory hallucinations with TMS. In patients, sLORETA revealed a decrease in alpha-2, beta-1 and -2 bands in the left hemisphere.
Lee et al. [86]	2006 Apr	SCZ1(with treatment refractory auditory hallucinations—AHs) = 25 SCZ2(without AH) = 23	SCZ1: 39.2 years, (M:F) = 11:14; SCZ2: 38.5 years, (M:F) = 10:13	Risperidone/olanzapine	LORETA, fast Fourier transformation (FFT)	Auditory stimuli	18 (Fp1, F3, C3, P3, Fp2, F4, C4, P4, F7, T3, T5, O1, F8, T4, T6, O2, T1, T2)	Patients with auditory hallucinations (AHs) had more left hemisphere activity on Bins 1 and 2 in the anterior region. In patients without AH and controls, these activities were on average the same. Increased beta-1 activity in BA40 (left inferior parietal lobule) and beta-2 activity in BA10 (left medial frontal gyrus) was revealed in patients with AH.
Itoh et al. [71]	2011 Aug	SCZ (first episode) = 17, CTL = 17	SCZ: 26.5 years, (M:F) = 11:6; CTL: 26.5 years, (M:F) = 11:6	Drug-free	LORETA	No data	19 (FP1, FP2, F3, F4, F7, F8, C3, C4, P3, P4, O1, O2, T3, T4, T5, T6, Fz, Cz, and Pz)	Individuals in group SCZ had higher delta-band activity. The total SANS score showed a negative correlation with LORETA values for delta-band activity in the right frontal gyrus and right parahippocampal gyrus.
Lehmann et al. [87]	2005 Feb 28	SCZ (first episode) = 7, CTL = 27	SCZ: 23.9 ± 5.4 years, (M:F) = 18:9; CTL: 24.4 ± 4.5 years, (M:F) = 18:9	Drug-free	LORETA	Different paradigms	19	Using k-means, EEG data were categorized into four groups of microstates. Only in class B did the topography change; SCZ mainly showed more activity on the left side and in the anterior part of the body. The concatenation of microstates (syntax) was often disturbed in patients.
Higuchi et al. [88]	2008 Apr	SCZ = 16, CTL = 16	SCZ:31.0 years, (M:F) = 5:11; CTL: 31.2 years, (M:F) = 5:11	Haloperidol/risperidone/perospirone/chlorpromazine/risperidone + perospirone/drug-free	LORETA	Auditory oddball paradigm	19 (FP1, FP2, F3, F4, F7, F8, C3, C4, P3, P4, O1, O2, T3, T4, T5, T6, Fz, Cz and Pz)	In areas including the left STG, left precentral gyrus, left middle frontal gyrus, and left precuneus, the P300 current source density was lower in group SCZ. After a 6-month course of olanzapine administration, a levodominant pattern of P300 current source density was observed, which was comparable to that in healthy subjects.
Horacek et al. [89]	2007	SCZ = 9	SCZ: 34.4 ± 9.1 years, (M:F) = 5:4	Olanzapine + levomepromazine + quetiapine + amisulpride + ziprasidone/risperidone + amisulpride or clozapine + olanzapine + haloperidol/lithium carbonate + sulpiride + levomepromazine + carbamazepine + risperidone + ziprasidone + carbamazepine + quetiapine + fluphenazine decanoate	Low-frequency rTMS, LORETA, 18FDG PET	10 days of TMS treatment	19	PANSS, HCS, AHRS showed statistically significant improvement. Transcranial magnetic stimulation increased brain metabolism in the frontal lobes and contralateral cortex while decreasing it in the left superior temporal gyrus and adjacent areas.
Sumiyoshi et al. [90]	2006 Sep 30	SCZ = 5, CTL = 5	SCZ: 40.2 ± 8.1 years, (M:F) = 4:1; CTL: 40.2 ± 8.1 years, (M:F) = 4:1	Olanzapine	LORETA, ERP (P300)	Oddball paradigm	19 (FP1, FP2, F3, F4, F7, F8, C3, C4, P3, P4, O1, O2, T3, T4, T5, T6, Fz, Cz, and Pz)	During olanzapine administration, scores on the AVLT, BPRS, and GAF questionnaires improved significantly. After patients switched to olanzapine, the P300 amplitude increased.
Saletu et al. [91]	2005 Apr	DS = 60, CTL = 20	DS: 51.1 ± 3.13 years, (M:F) = 0:60; CTL: 23–34 years, (M:F) = 10:10	Placebo, citalopram, imipramine	LORETA	Vigilance-controlled EEG (V-EEG) and a 4 min resting EEG (R-EEG)	19	In patients with depression, a decrease in the power of the theta and alpha-1 ranges and a decrease in the delta range in the BA13 and BA25 regions were shown. After citalopram treatment, an increase was observed in the beta-3, beta-2, beta-1, and alpha-2 ranges, especially on the right.
Tislerova et al. [92]	2008	SCZ = 41 (antipsychotic naive = 11, clozapine = 8, olanzapine = 10, risperidone = 12), CTL = 20	SCZ: 26.0 years, (M:F) = 22:19; CTL: 26.0 years, (M:F) = 10:10	Benzodiazepines/antidepressants/anticholinergics/mood stabilizers/drug-free	LORETA	Acoustic stimulation	19 (Fp1, Fp2, F7, F3, Fz, F4, F8, T3, C3, Cz, C4, T4, T5, P3, Pz, P4, T6, O1 and O2)	Patients receiving clozapine showed increased delta and theta frequencies in the anterior cingulate and medial frontal cortex and decreased alpha-1 and beta-2 frequencies in occipital regions. And patients taking olanzapine showed increased theta frequencies in the anterior cingulate, decreased alpha-1, beta-2 and beta-3 frequencies in the occipital cortex and posterior limbic structures, and decreased beta-3 frequencies in the frontotemporal cortex and anterior cingulate.
Molina et al. [72]	2019 Jun	SCZ = 38 (first episodes = 17, stable chronic = 21), CTL = 53	SCZ: 32.77 years, (M:F) = 22:16; CTL: 31.43 years, (M:F) = 29:24	Atypical antipsychotics/serotonin reuptake inhibitors/benzodiazepines	sLORETA, MRI (T1)	Oddball 3-stimulus paradigm	33 (Fp1, Fp2, F7, F3, Fz, F4, F8, FC5, FC1, FCz, FC2, FC6, T7, C3, Cz, C4, T8, TP9, CP5, CP1, CP2, CP6, TP10, P7, P3, Pz, P4, P8, PO9, PO10, O1, Oz, O2)	In frontal and parietal regions, control participants showed greater differences in P3b sources than patients. The activation of P3b sources was reduced in S patients in the medial frontal and anterior cingulate gyrus (BA9, BA32, BA33), lateral prefrontal (BA9, 10, 11), and orbital frontal gyrus (BA11, BA25).
González-Hernández et al. [93]	2014 Oct	SCZ = 48, CTL = 55	SCZ: 30.66 ± 9.59 years, (M:F) = 18:31; CTL: 32.20 ± 9.29 years, (M:F) = 25:30	All medicated	srVEP (C1, N1, P1), LORETA, Z-transformation		58 monopolar	Disturbances in the thalamus, posterior cingulate, precuneus, superior parietal, and medial occipitotemporal regions were related to symptom intensity. Positive sensations were significantly associated with sensory processing deficits (P1), whereas negative symptoms were significantly associated with perceptual processing difficulties (N1).
Kleinlogel et al. [69]	2007 Dec	SCZ (first episode) = 18, CTL = 18	SCZ:25.0 ± 4.9 years, (M:F) = 15:3; CTL: 24.6 ± 4.3 years, (M:F) = 15:3	Haloperidol/risperidone/olanzapine/quetiapine/quetiapine + haloperidol/solianol + haloperidol/drug-free	LORETA, independent component analysis (ICA), ERP (P300)	Visual task	21	Patients had an increased level of NGA compared to healthy controls. SCZ patients showed a decreased amplitude of P300 response to both Go- and NoGo stimuli and an increased P300 latency to NoGo stimuli.
Luckhaus et al. [94]	2013	SCZ = 18, BPE = 1	35.3 ± 8.2 years, (M:F) = 19:0	Not stated	sLORETA, EOG, independent component analysis (ICA), ERP (P100, N170, N250)	CPT paradigm	28	Training of Affect Recognition (TAR) was used to detect decreased activity in the parieto-temporo-occipital regions of the left hemisphere at 172 milliseconds and increased activation in the right dorsolateral prefrontal cortex and anterior cingulate at 250 milliseconds.
Lehmann et al. [95]	2014 Aug 20	SCZ (first episode) = 30	(M:F) = 18:12	Drug-free	eLORETA, conjunction analysis, principal functional connectivity	Resting with closed eyes	Bern and Berlin 19 electrodes (Fp1/2, F7/8, F3/4, Fz, C3/4, Cz, T3/T4, T5/6, P3/4, Pz, O1/2); 21 electrodes Fpz and Oz in addition) in Berlin	A decrease in overall brain activity in the cortex, especially in the anterior cingulate and temporal lobes, has been demonstrated in the group SCZ. There is evidence of varied and frequency-dependent increases in brain activity in specific areas in individuals with schizotypal personality disorder and depression.
Mientus et al. [96]	2002 Nov 30	SCZ = 19, Sh = 19, D = 30, CTL = 20	SCZ: 35.58 ± 10.69 years, (M:F) = 10:9; CTL: 36.15 ± 10.09 years, (M:F) = 10:10	Drug-free	LORETA	No data	19	In SCZ, cortical hypoactivation is noted in the anterior cingulate and temporal lobes. Schizotypal personalities and depressed patients show signs of a complex, frequency-dependent pattern of hyperactivation in parietal areas.

**Table 2:** Summary of studies exploring the effects of various treatments on schizophrenia patients. (NGA*—NoGo-anteriorization).

Method of Treatment of the Patient	Number of Studies	Changes in Electrical Activity
Olanzapine	5	(1) Enhancement in P300 current (2) Rise in P300 amplitudes (3) Rise in theta frequencies in the anterior cingulum (4) Decline in the occurrence of alpha-1, beta-2, and beta-3 waves in the occipital brain and posterior limbic areas (5) Decrease in the presence of beta-3 frequencies in the frontotemporal cortex and anterior cingulum (6) Reduced NGA*
Clozapine	3	(1) Rise in delta and theta frequencies in the anterior cingulate and medial frontal cortex (2) Along with a decline in alpha-1 and beta-2 frequencies in the occipital regions (3) Reduced NGA*
rTMS	1	(1) Reduced NGA* OR (2) No significant changes were found between non-antipsychotic users and risperidone users
Risperidone	4	There is a lack of data regarding influence
Levomepromazine	1	There is a lack of data regarding influence
Quetiapine	1	There is a lack of data regarding influence
Amisulpride	1	There is a lack of data regarding influence
Ziprasidone	1	There is a lack of data regarding influence
Haloperidol	2	There is a lack of data regarding influence

## Supplementary Materials

The following supporting information can be downloaded at: https://www.mdpi.com/article/10.3390/jcm13175108/s1, Table S1: Characteristics of the included studies [105,106,107,108,109,110,111,112,113,114,115,116,117,118,119,120,121,122,123,124,125,126,127,128,129,130,131,132,133,134,135,136,137,138,139,140,141,142,143,144].
