# Supplementary material for: EEG Techniques with Brain Activity Localization, Specifically LORETA, and Its Applicability in Monitoring Schizophrenia
Source: J Clin Med. 2024 Aug 28;13(17):5108. doi: 10.3390/jcm13175108 (PMC11395834; doi:10.3390/jcm13175108)
Supplement: Supplementary file 1 [file jcm-13-05108-s001.zip › jcm-3102998-supplementary.pdf]

Table S1. Characteristics of the included studies.

Study	Date	Sample size	Population characteristics	Paradigm	EEG data processing	EEG sensors	EEG Add-ons	Results
Bott et al. [105]	2023 Apr 19	H=40	23.4 ± 2.9 years, (M:F)=19:21	Probabilistic cueing paradigm, 2 types of stimuli and 2 types of visual cues with painful sensaitions	MATLAB	64	Not implemented	Pain stimulus intensity and anticipation affect pain assessment, local brain activity, and connectivity between brain regions.
Curot et al. [44]	2022 Sep 12	DRE=9	42.1 ± 14.1 years, (M:F)=7:2	EEG for 5-12 days, search for interictal fast ripples	Klusters software, SpyKING CIRCUS	64 (implanted, in tetrodes)	SEEG, MRI, CT	Neuronal activation was higher during fast ripples after which suppression of neuronal activity was shown in local networks.
Rho et al. [106]	2023 Jan 24	H=26	Right-handed, 26 ± 3 years, (M:F)=18:8	Eyes-closed 4-minute resting, a 3-minute Cold Pressor Test (CPT) task, and a 3-minute resting	EEGLAB, independent component analysis (ICA), Adaptive Mixture Independent Component Analysis (AMICA) algorithm, PCA, MVAR model	128	Not implemented	Differences in Directed Transfer Function (dDTF) were found between nodes at different frequencies and times, both at rest and during CPT.

Paban et al. [57]	2018 Oct 17	H=32	32 ± 8.8 years, (M:F)=19:13	Resting	EEGLAB, independent component analysis (ICA), BRAPH software, PCA	64	eLORETA	Two passive coping strategies, involving social support and avoidance, and two active coping strategies, namely problem solving and positive thinking, were identified. In the resting state, passive coping-individuals were highly involved in processes that were in the delta range. Subjects using active coping strategies showed a limited number of areas restricted to the temporal lobe in the theta and alpha bands

Ehrlich et al. [107]	2023 Jul 25	CTL=22, LD=24	Not found	Symptomatic speech and asymptomatic tasks such as whispering and writing	Not found	128	Not implemented	Symptomatic speech exhibited in patients with LD showed changes in the prefrontal-parietal loop, with hyperfunctional connectivity between the left middle frontal gyrus and the right superior parietal lobe hinged on the age of onset and symptom duration.
Vartanov A.V. [108]	2023 Dec 6	E=1	31 years	Resting	Brainsys	19	Not implemented	A new "Virtually implanted electrode" method has been experimentally confirmed in a patient with epilepsy, which quite accurately determines the spatial localization of electrical potentials.

Jaroszynski et al. [45]	2022 Aug 8	DRE=50	Not stated	Counting and listing word series	Region Of Interest (ROI) approach, high-frequency activity modulation (HFA) approach	128 (implanted)	SEEG, MRI, CT	Illusions involved the frontoparietal and anterior cingulate gyrus, were generally associated with lateral regions, and hallucinations were manifested posteromedially in the temporal lobe, but overall both perceptions were found at all levels of sensory activity.
Williams et al. [109]	2023 May 15	DRE=67	Males and females, 17 ± 3 years	Eyes-closed resting	MATLAB, Brain Connectivity Toolbox, Phase locking value (PLV) method	192	SEEG	Modules in the brain's phase synchronization connectomes form functional systems at multiple scales and frequencies.
Nguyen et al. [46]	2018 Jun 30	Not stated	Not stated	Visual stimuli of unpleasant and pleasant images of human faces	General Linear Model	64	fMRI	A combined approach including EEG and fMRI allows us to take into account temporal and spatial aspects of brain activation.

Hüning et al. [50]	2018 May 22	PI=38	Preterm infants born at the University Hospital Essen from March 2011 to December 2012, with a mean gestational age (GA) of 28.2 ± 2.3 weeks and a mean birth weight of 1093 ± 404 g	Recording within the first 72h of life	Burdjalov scoring system	4 (C3, P3, C4, and P4)	aEEG, MRI	A reduction in the deep gray matter volume was connected with a low Burjalov scale score on day 3 and on days 1-3.
Fan et al. [58]	2022 Apr 9	H=20	22.8 ± 1.7 years, (M:F)=12:8	Virtual reality technology	Not found	Not found	eLORETA	Fostering the activity of the mirror neuron system, visual reappearance of one's actions strengthens the functional integration of this zone with the sensorimotor cortex.
Coronel-Oliveros et al. [110]	2021 Feb 18	Not stated	Not stated	11 min real-time recordings	Modified Jansen & Rit neural mass model, Euler–Maruyama method, Python, functional connectivity	Not found	fMRI	An increase in inhibitory gain or β coefficient was associated with an increase in integration in the global dynamics of the neural network.
Kuzovkin et al. [111]	2020 May 12	DRE=100	33.18 ± 10.13 years, (M:F)=51:49, recruited from Neurological Hospitals in	Visual recognition task	Random Forest classification model	120 (implanted)	MRI	Visual cortex and inferior temporal areas are involved in automatic perceptual categorization.

			Grenoble and Lyon (France)					
Taylor et al. [112]	2020 Jan 5	H=21	Not stated	The oddball paradigm, sinusoidal tones	MATLAB	64	MEG	A set of tools for visualising M/EEG data has been developed to produce graphical representations of the scalp map and a three-dimensional construct of discrete clusters with local peaks.
Bernhard et al. [113]	2022 Nov	DRE=7	35 years, (M:F)=5:2, admitted to Maastricht University Medical center	Recording 22 hours including one period of sleep	MATLAB, Fieldtrip toolbox, Sleeptrip toolbox	19	MRI, EOG	Spindle duration and amplitude did not differ between fast and slow spindles. In the anterior thalamus, T2 spindles were more often single, and in T3 spindles were both single and bilateral, occurring in multiple channels

Li et al. [48]	2018 Dec 3	CTL=8, mAD=6	CTL: 62.75 ± 8.21 years, (M:F)=6:2, recruited from the local community; mAD: 72.5 ± 7.34 years, (M:F)=2:4, recruited from a local hospital	Verbal digit span task	Phase Lag Index (PLI), functional connectivity, functional regions of interest (ROIs)	32	fNIRS	Two major peaks were identified, one with possible P300 components at around 200-300 ms and the other related to the cognitive task at 1100 ms, which was significantly reduced in patients with Alzheimer's disease.
Pinti et al. [49]	2021 Feb 17	H=13	31 ± 8 years, (M:F)=9:4	Visual stimulation task and rest blocks	MATLAB, EEGLAB, Psychtoolbox, power spectral density (PSD) in the gamma band	26	bNIRS	Cross-correlation of bNIRS and EEG showed a negative correlation of EEG oxCCO during right-sided stimulation and a weaker correlation during left-sided stimulation, subsequently changing to a positive correlation.
Carboni et al. [114]	2020 Jul 6	CTL=16, E=49	CTL: 26 years, (M:F)=10:6; E: 31 years, (M:F)=25:24, from the Geneva University Hospital	Wakefulness with eyes open	Regions of interest (ROIs), high-dimensional tv-multivariate autoregressive (MVAR) models	128 or 256	MRI	The efficiency of the somato-motor network, ventral attention network, and default mode network was higher in all patients with TLE and ETLE as well as TLE with hippocampal sclerosis.

Kassab et al. [115]	2017 Oct 23	H=12	Between 13–27 years, (M:F)=6:6	Pedalling on an exercise bike, or during a cognitive task	MATLAB, VEPs	32	fNIRS	A prototype system for recording brain signals using fNIRS and EEG during visual stimulation and language tasks has shown potential for patients with stroke and epilepsy.
Ghaderi et al. [116]	2019 May 14	H=24	24.6 ± 3.97 years, (M:F)=12:12	Arithmetic and logical puzzle	MATLAB, adjacency matrix and connectivity index	19	Not implemented	During problem solving, arousal and excitation change, which is sporadically correlated with changes in connections between brain regions in the alpha and beta bands.
Arabadzhiyska et al. [117]	2022 Nov 30	H=31	White, between 18-35 years, (M:F)=12:19, recruited through The University of Glasgow subject pool	150 photorealistic images of faces during a simulated economic game with social and non-social contexts	MATLAB, PCA, Weibull function, linear discriminant analysis (LDA)	64	fMRI	Activity in the posterior frontal cortex (pMFC) was associated with neural activity in decision-making areas.

Takacs et al. [60]	2020 Mar 9	H=27	23.4 ± 3.3 years, (M:F)=9:18	S–R task	MATLAB, Brain Vision Recorder 1.2 software, Watts–Strogatz model, residue iteration decomposition (RIDE)	60	sLORETA	The amplitude of the P3 component decreased during the transition from conditions without overlap to complete overlap of features in BA40, BA6, and BA9.
Treder et al. [52]	2021 Dec 8	CTL=20, DRE=11	CTL: 25.01 years, (M:F)=12:8, DRE: 34.45 years, (M:F)=6:5, 10 from the Queen Elizabeth Hospital and 1 from La Paz University Hospital	712 images and identifing each as an object or a scene by pressing a button	MATLAB, FieldTrip, MVPA-Light, linear discriminant analysis (LDA)	128	iEEG, sMRI	An increase in the power of gamma rhythms was associated with successful memory in the hippocampus, which was accompanied by a decrease in the power of alpha rhythms.
Jami et al. [118]	2022 Sep 29	H=28	27 ± 5.3 years, (M:F)=22:6	IVA-CPT	MATLAB, EEGLAB, Horn and Schunck method, independent component analysis (ICA)	19	Not implemented	The proposed approach of EEG analysis using topocards made it possible to accurately assess types of attention such as focus, exposure, and comprehension.

He et al. [119]	2021 Oct 6	PD=7, MSA=4	PD: 65.45 ± 8.51 years, (M:F)=7:0; MSA: 11.95 ± 8.53 years, (M:F)=4:0	Following the movements of a cartoon man	MATLAB, imaginary coherence (IC), hidden Markov model and K-medoids	10 (DBS, implanted)	MRI, CT	1. In PD patients, activity in the wide beta range in PPN and EEG was reduced when standing compared to sitting. 2. In patients with MSA, activity in the narrow beta range in PPN and EEG was lower when walking compared to sitting.
Mohan et al. [61]	2022 Mar	CTL=10, T=10	CTL: 27 ± 4.71 years, (M:F)=4:6; T: 25.9 ± 5.49 years, (M:F)=8:2	Auditory paradigm	MATLAB, EEGLAB, ERPLAB, functional connectivity	64	Not implemented	In the tinnitus group, the ERP amplitude over pgACC/vmPFC, dACC, PCC, primary auditory cortex and parahippocampus was reduced and the amplitude in the frontal and parietal regions was increased.
Das et al. [51]	2021 Oct 20	N=36	39 ± 1.4 weeks, (M:F)=21:15	Resting	MATLAB, wavelet transform coherence analysis; near-infrared spectroscopy	8 (C3, C4, P3, P4, O1, O2, Cz, and Fz)	aEEG, sMRI, NIRS-SctO2	WTC analysis of neurovascular coupling predicted brain damage in neonates with hypoxic-ischaemic encephalopathy with a sensitivity of 69% and specificity of 90%.

Wang et al. [120]	2019 Aug 1	H=16	25.4 years, (M:F)=4:12	Visuotactile matching paradigm	MATLAB, EEGLAB, Phase locking value (PLV) method, COSTRAP algorithm, phase coupling, phase-amplitude coupling	126	Not implemented	The network associated with target detection was observed in the alpha and beta frequency bands (~10 and ~20 Hz, respectively) and activated the right hemisphere. The second network was in the beta band (~20 Hz), activating areas of the left hemisphere. The third network was observed in the theta band (~5 Hz) and activated frontal regions. The fourth network, spatially similar to the first, showed reduced cross-frequency connectivity
Ertl et al. [62]	2021 Feb 3	H=23	25.3 ± 5.0 weeks, (M:F)=11:12	6-degree-of-freedom motion platform	MATLAB, independent component analysis (ICA)	32	MEG, eLORETA	Activity in BA 40, an area related to spatial attention and reorientation, decreased when head position did not match eye position or direction of movement.

Pei et al. [8]	2022 Sep 30	Not found	Not found	Auditory, visual and combined modalities of a sequence of short Chinese sentences	Not found	Not found	TMS	Using continuous theta stimulation, it was shown that the left prefrontal cortex is activated during the integration of Chinese sentences.
Galinsky et al. [47]	2018 Apr 13	Not found	Not found	Resting	CORT-JESTER, Maxwell's equations, frequency-dependent inverse Green function	32 or 64	rs-fMRI, MEG	The fusion of audio-visual information takes place across all levels of linguistic units, with various brain regions engaged in this process at different levels.
Völker et al. [59]	2021 Feb 19	H=21	25.0 ± 2.6 years, (M:F)=7:14	Vertical bar image	EEGLAB, Bland–Altman (BA) analysis	64	eLORETA	None of the studies reviewed for CSD assessments during pain provided sample size calculations, and fewer than 20% reported absolute measures of central tendency and variance. The CSD results showed high reliability without heteroscedasticity and systematic bias. Reliability is particularly high in the alpha band at resting state.

Del Río M et al. [121]	2018 Aug 1	H=24	21.38 years, (M:F)=5:19	Variation of the Porta test	MATLAB, Psychophysics Toolbox, Infomax independent components analysis (ICA), Phase locking value (PLV) method	64	Not implemented	In bCFS tests, theta phase reset and synchronization occur before suppression is stopped.
Siebenhühner et al. [122]	2020 May 6	H=19	Not stated	Eyes-closed rest	Phase locking value (PLV) method, cross-frequency coupling (CFC), phase–amplitude coupling (PAC)	192	SEEG, MRI, CT, MEG	1. Genuine inter areal CFC is present in human RS activity in both SEEG and MEG data. 2. CFS and PAC networks had distinct spectral patterns and opposing distribution of low- and high-frequency network hubs, implying that they constitute distinct CFC mechanisms. 3. These results provide evidence for interareal CFS and PAC being 2 distinct mechanisms for coupling oscillations across frequencies in large-scale brain networks

Nguyen et al. [123]	2019 Feb 22	H=20	Not found	Emotion processing and regulation tasks	Not found	Not found	fMRI	The main source of the emotion-related brain network was the ventrolateral prefrontal (VLPFC) cortex, and emotional shifts were associated with condition-specific interactions
Weber et al. [124]	2021 Jan 1	H=12	24.33 ± 0.99 years, (M:F)=5:7	Sleeping	MATLAB, Discrete Fourier Transform (DFT)	6	MEG	1. There was an elevation in activity in low gamma (30–40 Hz) and neighboring beta range (<30 Hz) during spindles. 2. The infra-low rhythm of the ball is characterized by a low power of the gamma range, the frequency is higher than than that of spindle activity. 3. Spindles assist in the process of processing local memory between distributed networks

Gong et al. [6]	2018 Nov 11	H=40	20 ± 2 years, (M:F)=40:1	Resting state and shooting sessions	MATLAB, EEGLAB, Phase locking value (PLV) method	16	Not implemented	1. A negative correlation was found in the Beta 1 and Beta 2 ranges between performance during shooting and the functional connection between the prefrontal, frontal and temporal regions of the right hemisphere of the brain. 2. During the shooting, lower functional connectivity was observed among the shooters with the best results, as well as high efficiency of global integration and low efficiency of local information integration

Handiru et al. [53]	2021 July 26	CTL=15, TBI=17	CTL: 47 ± 12.8 years, (M:F)=8:7; TBI: 48.7 ± 12.5 years, (M:F)=13:4	Dynamic posturography platform	EEGLAB, functional segregation and integration analysis	64	MRI	1. During the task, there was a negative correlation between modality in the theta range and BBS in the group with TBI 2. Lower network connectivity in the beta range was revealed due to a violation of the structural integrity of the white matter. 3. In the TBI group, low equilibrium values for the displacement of the center of pressure during the task and on the Berg balance scale compared with the control, a decrease in brain activity and connectivity was also observed during the tasks

Xiong et al. [125]	2023 Jul 12	CTL=27, T=57	Not found	Not found	Not found	Not found	Not implemented	Patients showed marked activation in the auditory cortex (BA 21), and in the group with moderate to severe tinnitus there was enhanced connectivity between the parahippocampus and posterior cingulate gyrus and between the auditory cortex and the insula
Noel et al. [126]	2019 Aug 31	H=17	23.9 ± 3.7 years, (M:F)=7:10	The oddball paradigm	MATLAB	128	Not implemented	At the PPS boundary, when stimuli appear, the global strength of the neural response increases

Staresina et al. [127]	2023 July 10	H=10	39.9 years, (M:F)=5:5	Capturing neuronal firing (multiunit activity (MUA))	MATLAB, CCG analysis, Phase locking value (PLV) method	8 (implanted)	iEEG	1. During sleep, NREM communication during sleep and neural activity are controlled by interaction between SOs, spindles and ripples. 2. Spindles increase the appearance of ripples. 3. The synchronization of sleep rhythms throughout the MTL was revealed, and this in turn leads to interregional neural communication - the basis for system consolidation.
Bluschke et al. [63]	2018 Feb 27	CTL=35, ADHD=31	CTL: 12.55 ± 1.55 years, (M:F)=33:2; ADHD: 12.64 years ± 1.76, (M:F)=29:2	Stop-Change paradigm	Residue iteration decomposition (RIDE) analysis	60	Not implemented	Patients with ADHD show lower behavioral analysis results compared to the control group: more mistakes were made and long waiting periods with stimuli that indicated stopping or changing actions

Frot et al. [128]	2022 Oct 1	Not found	Not found	Emotional faces images	Not found	Not found	Not implemented	Early responses in anterior part of insula (AI) are associated with arousal and behavioral responses to emotional stimuli via pathways through the superior colliculus and dorsal pulvinar
Domínguez-Borràs et al. [129]	2019 May 31	E=7	30.29 years, (M:F)=3:4	Auditory, visual, and combinations of both stimuli	E-Prime, MATLAB	8 (implanted)	iEEG, MRI	1. The amygdala's response to frightening and neutral stimuli was different 2. No super- or subadditivity effects were found in any of the bimodal responses 3. Emotion processing from different stimuli occurs by a similar mechanism in the early stages 4. A more pronounced and different response to multisensory stimuli was revealed in the later stages of emotion formation

Ding et al. [130]	2022 Mar 7	H=20	22 ± 2.6 years, (M:F)=10:10	Memory paradigm	MATLAB, EEGLAB, independent component analysis (ICA), novel algorithm named Weighted K-order Propagation Number (WKPN), Phase locking value (PLV) method	64	Not implemented	1. A new experimental paradigm has been identified and tested 2. A new algorithm has been proposed the K-order propagation number algorithm.
Friedrich et al. [64]	2017 Jun 11	H=28	S1: N = 15, S2: N = 13; 24 years	Tactile GO/NOGO task	Residue iteration decomposition (RIDE) analysis, MATLAB	60	Not implemented	The S1 and S2 brain regions differ in their ability to trigger inhibition processes.
Ibáñez-Marcelo et al. [131]	2019 Jul 2	H=37	Highs: 23 ± 1.9 years, (M:F)=7:11; Lows: 22.7±1.5 years, (M:F)=9:10 (based on SSHS scale)	The visual or kinesthetic imagery of a rotated head position	MATLAB, EEGLAB, independent component analysis (ICA)	32	Not implemented	During sensory and imagery tasks when compared to basal conditions persistent homology demonstrates a lesser degree restructuring of EEG asset in individuals with highs group compared to those with lows group.

Nash et al. [65]	2023 April 1	H=93	21 years, (M:F)=38:55	Stroop task	MATLAB	64	sLORETA	The accuracy of reactions is impaired in an anxious state in the Stroop task and error-related neural processes, characterized by reduced activation of the dorsal anterior cingulate cortex and compensatory activation in the right lateral prefrontal cortex.
Rué-Queralt et al. [132]	2023 Oct 15	H=70	29.7 years, (M:F)=36:34	Visual paradigm, circular sine wave grating	MATLAB, EEGLAB, Locally Spherical Model with Anatomical Constraints (LSMAC)	256	MRI	Different information processing mechanisms are performed in different frequency ranges: spatially distributed activity is typical for low time frequencies (alpha and theta) and low graphical spatial frequencies, localized electrical activity is observed at high time frequencies (high and low gamma) at limited frequencies.

Gallina et al. [54]	2022 May 19	VFDL=14, VFDR=13, CTL1=9, CTL2=14	VFDL: 53.08 ± 2,58 years, (M:F)=10:4; VFDR: 58.9 ± 5,20 years, (M:F)=10:3; CTL1: 43.22 ± 9,65 years, (M:F)=5:4; CTL2: 54.29 ± 8,28 years, (M:F)=7:7	Eyes-closed rest	MATLAB, EEGLAB, CSD toolbox, independent component analysis (ICA), Principal Component Analysis (PCA)	59	sLORETA	At rest, oscillatory connections are selectively disrupted due to lesions of the posterior parts of the brain
Bednar et al. [133]	2018 Jul 24	H=33	21 years, (M:F)=11:22	Auditory stimuli + simple target detection task	MATLAB, EEGLAB, multivariate linear reconstruction model	128	MRI	Trajectory decoding is sensitive to both ILDs stimuli and ITD stimuli.
Iannotti et al. [134]	2020 Oct 14	DRE=10	17 years, (M:F)=4:6	Rest	BrainVision Analyser, MATLAB, BOLD method	64 or 256	fMRI	A positive correlation was found between the time intervals of dFC and VarIED
Chung et al. [135]	2018 May 18	CTL=15, PD=15	CTL: 62,53 ± 8.44 years, (M:F)=9:6; PD: 62 ± 10.87 years, (M:F)=9:6	Upper limb ballistic movement task	MATLAB, EEGLAB, independent component analysis (ICA), Brain Electrical Source Analysis (BESA)	128	fMRI	An increased level of desynchronization in the beta range (13-30 Hz), associated with movements, was found in the additional motor zone in patients not taking drugs with PD

Li et al. [66]	2019 Nov	CTL=12, SCA=12	CTL: 36.4 ± 11.6 years, (M:F)=7:5; SCA: 37.3 ± 11.6 years, (M:F)=7:5	Vocalizing the vowel sound and hearing their voice pitch unexpectedly shifted downward	NetStation, EEGLAB	64	fMRI	A meaningful rather large vocal compensation for pitch disturbances was observed in patients with SCA compared to the control group
Delis et al. [136]	2018 Mar 23	H1=15, H2=10	H1: 26 ± 2 years, (M:F)=9:6; H2: 24 ± 2 years, (M:F)=6:4	Task using Pantograph	EEGLAB, Principal Component Analysis (PCA), Independent Component Analysis (ICA), EEG2Beh(avior)	64	EMG	An active perception strategy based on sensorimotor mechanisms regulates the processes of perception and thinking, which ultimately affects decision-making
So et al. [137]	2018 Feb 1	S=7, CTL=8	S: 33.29±12.66 years, (M:F)=6:1; CTL: 32.50 ±8.26 years, (M:F)=5:3	Rest, Sternberg working memory task	Hamming window, fast Fourier transform, Phase locking value (PLV) method	66	MRI	There were no differences in behavioral indicators between the two groups

Peterson et al. [138]	2019 May 18	H=30	22.5 ± 4.8 years,(M:F)=15:15	Sensorimotor perturbations: a side-to-side pull at the waist and a 20-degree field-of-view rotation	EEGLAB Cleanline plugin	136	EMG	Cortical-cortical alpha coupling (8-13 Hz) between occipital and parietal regions was markedly reduced under the influence of standing rotation. The theta frequency (4-8 Hz) of the connection between the supplementary motor area and the central motor area was increased under the influence of percussive vibration while standing up
Wadhera et al. [55]	2020 Mar 4	ASD=30, CTL=30	ASD: 14.6 ± 3.2 years, (M:F)=25:5; CTL: 13.4 ± 2.5 years, (M:F)=18:12	0- and 2-back task	MATLAB, PsychToolbox	21	Not implemented	In ASD, behavioral indicators were reduced with an increase in cognitive load
Wolff et al. [67]	2017 Oct 12	H=26	24.62 ± 3.10 years, (M:F)=10:16	A cued-task-switching paradigm, ERP	Not found	60	sLORETA	Task switching effects were more prominent in the block where memory activation was required compared to the block where it was not required

Baumeister et al. [139]	2016 Sep 12	ADHD=16	11.5 years, (M:F)=12:4	Combined Flanker/NoGo task	Not stated	Not stated	fMRI	There was a decrease in ADHD symptoms and an increase in activity in areas associated with inhibition control in patients from the NF group compared with the control group
Laventure et al. [140]	2018 Aug 21	H1=25, H2=24	H1: 24.8 ± 5.0 years, (M:F)=14:11; H2: 24.9 ± 4.0 years, (M:F)=13:11	Motor sequence learning (MSL) paradigm, sleep periods.	MATLAB	10	Not implemented	During sleep, carotid spindles are associated with reactivation of recently acquired motor sequence traces in memory. Low frequencies increase connectivity throughout the cerebral cortex
Ratcliffe et al. [141]	2022 May 23	H=28	22.64 ± 3.95 years, (M:F)=10:18	A delayed-match-to-sample (DMS) task and an n-back task	MATLAB, Fieldtrip, MVPA-light, Psychophysics Toolbox, Rstudio	128	Not implemented	An increase in fronto-medial theta (FMT) power was found during the n-back task compared to the DMS task
Morales et al. [142]	2018 Oct 19	Not found	Not found	Not stated	Finite difference method in anisotrophic media (AFDM), Maxwell's equations	128	MRI	Differential head model methodology improves the accuracy of head structure modeling for brain activity imaging

Agrawal et al. [143]	2019 Mar 7	H=20	24 years, (M:F)=13:7	EEG-fMRI, graph theory.	BOLD method	32	fMRI	The nature of task-related stimuli influenced the activation and deactivation of the default mode network (DMN), which was also related to neuronal activity during the task
Song et al. [144]	2021 Aug 15	H=25	22.5 years, (M:F)=10:15	short unmanned aerial vehicle (UAV) video clips, ICA, ERP	Brainstorm software, template ICBM152	64	Not implemented	P3 like-component can be caused by dynamic distractors

(H - healthy participants, DRE - drug-refractory epileptic patients, LD - laryngeal dystonia, PI - preterm infants, mAD - mild Alzheimer's disease, E - epileptic patients, PD - Parkinson's disease, MSA - multiple system atrophy, T - participants with tinnitus, N - neonates, VFDL - visual field defect due to lesions to the left posterior cortices, VFDR - visual field defect due to lesions to the right posterior cortices, ADHD - Attention Deficit Hyperactivity Disorder, SCA - spinocerebellar ataxia, S - schizophrenia, ASD - Autism Spectrum Disorder, TBI - Traumatic brain injury, CTL - controls).
